# Effect of a small molecule inhibitor of nuclear factor-κB nuclear translocation in a murine model of arthritis and cultured human synovial cells

**DOI:** 10.1186/ar1834

**Published:** 2005-09-30

**Authors:** Kyoko Wakamatsu, Toshihiro Nanki, Nobuyuki Miyasaka, Kazuo Umezawa, Tetsuo Kubota

**Affiliations:** 1 Department of Microbiology and Immunology, Tokyo Medical and Dental University Graduate School of Health Sciences, Tokyo, Japan; 2 Department of Medicine and Rheumatology, Tokyo Medical and Dental University Graduate School, Tokyo, Japan; 3 Department of Applied Chemistry, Keio University, Kanagawa, Japan

## Abstract

A small cell-permeable compound, dehydroxymethylepoxyquinomicin (DHMEQ), does not inhibit phosphorylation and degradation of IκB (inhibitor of nuclear factor-κB [NF-κB]) but selectively inhibits nuclear translocation of activated NF-κB. This study aimed to demonstrate the antiarthritic effect of this novel inhibitor of the NF-κB pathway *in vivo *in a murine arthritis model and *in vitro *in human synovial cells. Collagen-induced arthritis was induced in mice, and after onset of arthritis the mice were treated with DHMEQ (5 mg/kg body weight per day). Using fibroblast-like synoviocyte (FLS) cell lines established from patients with rheumatoid arthritis (RA), NF-κB activity was examined by electrophoretic mobility shift assays. The expression of molecules involved in RA pathogenesis was determined by RT-PCR, ELISA, and flow cytometry. The proliferative activity of the cells was estimated with tritiated thymidine incorporation. After 14 days of treatment with DHMEQ, mice with collagen-induced arthritis exhibited decreased severity of arthritis, based on the degree of paw swelling, the number of swollen joints, and radiographic and histopathologic scores, compared with the control mice treated with vehicle alone. In RA FLS stimulated with tumor necrosis factor-α, activities of NF-κB components p65 and p50 were inhibited by DHMEQ, leading to suppressed expression of the key inflammatory cytokine IL-6, CC chemokine ligand-2 and -5, matrix metalloproteinase-3, intercellular adhesion molecule-1, and vascular cell adhesion molecule-1. The proliferative activity of the cells was also suppressed. This is the first demonstration of an inhibitor of NF-κB nuclear translocation exhibiting a therapeutic effect on established murine arthritis, and suppression of inflammatory mediators in FLS was thought to be among the mechanisms underlying such an effect.

## Introduction

Rheumatoid arthritis (RA) is a chronic inflammatory disease that affects nearly 1% of the population worldwide and can lead to significantly impaired quality of life. Mortality rates are also significantly increased in patients with RA, and currently available therapies are often unable to change the course of the disease; therefore, further improvements in therapy are required. In this regard the recent application of biologic agents such as monoclonal antibodies to tumor necrosis factor (TNF)-α and IL-6 receptor, and recombinant soluble TNF-α receptor have been of great interest. Many cytokines, chemokines, adhesion molecules and matrix degrading enzymes have been demonstrated to play a role in synovial proliferation and joint destruction, which are the main pathologic features of RA. Notably, the efficacy of these biologic agents has indicated that intervention in a single cytokine pathway can achieve significant suppression of the complex inflammatory network and ameliorate disease. However, there are negative aspects to therapy with biologic agents, such as opportunistic infections, infusion reactions, high cost, and the fact that there are some patients in whom RA remains active regardless of the use of biologics. Therefore, further development of small molecular agents that specifically interrupt the critical intracellular pathways that are activated in RA synovium could prove beneficial.

The transcription factor nuclear factor-κB (NF-κB) forms a heterodimer or a homodimer of the subunit members, and in the cytoplasm of unstimulated cells it binds to natural inhibitors of NF-κB (IκB), which prevent it from entering the nucleus. The most common activated form of NF-κB in inflammatory cells consists of a p65 subunit and a p50 or p52 subunit [1-3]. In synovial tissue from patients with RA, p65 and p50 have been shown to be present in the nuclei of macrophage-like synoviocytes, fibroblast-like synoviocytes (FLS), and vascular endothelial cells, and probably play a pivotal role in the pathogenesis of RA [4-7]. The cytokines IL-1 and TNF-α activate and can be activated by NF-κB, and this positive regulatory loop amplifies the expression of other cytokines, chemokines, adhesion molecules, and enzymes in inflamed tissue [2]. Therefore, NF-κB should be considered a primary target for new types of anti-inflammatory treatments. Indeed, several recent studies have already shown significant effectiveness of this strategy. For example, *in vivo *experiments using murine arthritic models that employed intra-articular adenoviral gene transfer of dominant negative IκB kinase β [8] or super repressor IκBα [9], or alternatively intra-articular injection of NF-κB decoy oligonucleotides [9,10] demonstrated decreased severity of joint swelling. Moreover, *ex vivo *adenoviral gene transfer of IκBα into human synovial tissue inhibited the expression of inflammatory mediators [11]. Apart from gene transfer techniques, intravenous injection of a chimeric protein comprising the super-repressor IκBα fused to the membrane-transducing domain of the HIV Tat protein was shown to be effective in a rat model of acute pleuritis, although arthritis was not addressed in that study [12].

Only a limited number of studies testing the *in vivo *effects of small molecular weight compounds on arthritis have been reported [13]. These include a proteasome inhibitor PS-341 [14], and IκB kinase inhibitors BMS-345541 [15] and SPC 839 [16], which improved clinical and pathologic findings in murine arthritis. Another NF-κB inhibitor designated SP100030 was also shown to suppress collagen-induced arthritis (CIA) [17], but it appeared to be less efficient, possibly because it selectively affects T cells and not fibroblasts or endothelial cells. Recently, a peptide inhibitor of NF-κB that blocks association of NEMO (NF-κB essential modulator) with IκB kinases has been shown to ameliorate carrageenan-induced mouse paw inflammation, CIA, and RANKL (receptor activator of NF-κB ligand)-induced osteoclastogenesis [18,19].

Because RA is a chronic systemic disease, low molecular weight, cell-permeable agents that can block the NF-κB pathway with high specificity – if they become available – may have an advantage over gene transfer methods. In our search for such an inhibitor, we designed a compound named dehydroxymethylepoxyquinomicin (DHMEQ) using the parent structure of the antibiotic epoxyquinomicin C. We demonstrated that DHMEQ inhibits TNF-α-induced nuclear translocation of NF-κB, and does not inhibit phosphorylation and degradation of IκB, or a c-Jun N-terminal kinase (JNK) and a caspase-activating pathway in Jurkat T cells [20,21]. Here, we extended our study to test the therapeutic effect of DHMEQ on CIA, and to test the efficacy on the inhibition of the inflammatory pathway in human RA FLS. The results showed that this unique inhibitor of NF-κB nuclear translocation may hold promise for treatment of RA.

## Materials and methods

### Inhibitor of nuclear factor-κB

DHMEQ was synthesized as described previously [22]. It was dissolved in 100% dimethyl sulfoxide (DMSO) at 20 mg/ml and kept in aliquots at -30°C. Before use in cell culture, it was diluted with the medium described below to a final DMSO concentration of 0.05% or less, at which no effect of DMSO *per se *on NF-κB activity was observed.

### Induction of collagen-induced arthritis

Animal experiments were approved by the Institutional Animal Care and Use Committee of Tokyo Medical and Dental University. Male 8-week-old DBA/1J mice were purchased from Oriental Yeast (Tokyo, Japan). Bovine collagen type II (Collagen Research Center, Tokyo, Japan) was dissolved in 50 mmol/l acetic acid at 4 mg/ml and emulsified in an equal volume of Freund's complete adjuvant (Difco Laboratories, Detroit, MI, USA). Mice were immunized intradermally with 100 μl of the emulsion at the base of the tail. After 21 days (day 0) the same amount of the antigen emulsified in the same adjuvant was intradermally injected at the base of the tail as a booster immunization. By day 5, 20 out of the 25 mice developed signs of arthritis and were randomly allocated to two groups of 10 mice each: an experimental group and a control group. From days 5 to 18, 100 μg DHMEQ (5 mg/kg body weight) dissolved in 50 μl of 100% DMSO was injected subcutaneously every day into the inguinal region of the mice in the experimental group. Mice in the control group received 50 μl DMSO, injected similarly.

### Assessment of arthritis

The thickness of each hind paw was measured using a pair of digital slide calipers by an investigator who was blinded to the treatment groups. Out of 16 joints in each hind paw (i.e. the ankle, midfoot, first to fifth metatarsophalangeal [MTP] joints, interphalangeal joint of the first toe, and second to fifth proximal and distal interphalangeal joints), the swollen joints were identified using magnified pictures taken with a digital camera and counted. Radiographic assessment of bilateral second to fourth MTP joints was carried out using the following scoring systems: for soft tissue swelling 0 = not obvious, 1 = mild, 2 = marked; and for bone erosion 0 = not obvious, 1 = erosion < 0.3 mm in diameter, 2 = erosion > 0.3 mm in diameter. Use of this system yields a possible score between 0 and 12 per animal for each item. The left hind paw of each mouse was dissected, formalin-fixed, decalcified, embedded in paraffin, and stained with hematoxylin and eosin. Synovitis and bone destruction around the MTP joints of the sections were scored as follows: synovitis 0 = not obvious, 1 = synovitis < 0.2 mm at maximum thickness, 2 = synovitis > 0.2 mm at maximum thickness; bone destruction 0 = not obvious, 1 = obvious bone erosion, 2 = marked bone erosion associated with penetration of pannus into the marrow space. Radiographic and histopathologic assessments were performed by five investigators who were blinded to the assignment of mouse groups.

### Cell cultures

RA FLS lines were established, as described previously [23], from the synovial tissues of RA patients obtained at surgery. RA patients fulfilled the American College of Rheumatology criteria. All procedures involving human tissues were approved by the Ethics Committee of Tokyo Medical and Dental University, and consent forms were obtained from the patients involved in the study. The cells were cultured in 100 mm dishes with Dulbecco's modified Eagle's medium (high glucose) containing 10% heat-inactivated fetal calf serum (FCS; Givco, Rockville, MD, USA) and antibiotics. Cells were passaged between four and eight times and were used when the cultures had reached about 80% cell layer confluence.

### RT-PCR

RA FLS in 100 mm dishes were starved for 16 hours in medium without FCS, and then 10 μg/ml DHMEQ or vehicle was added. Twenty minutes later they were stimulated with 5 ng/ml TNF-α (PeproTech, London, UK) for 30 minutes, washed with phosphate-buffered saline (PBS), and detached from the dishes by treatment with trypsin-EDTA. Total RNA was isolated using RNeasy Mini Kit (Qiagen, Valencia, CA, USA) and treated with DNase I (Takara, Ohtsu, Japan), and RT-PCR was carried out using OneStep RT-PCR Kit (Qiagen) and the following primers: β-actin (5'-GTCCTCTCCCAAGTCCACACA, 3'-CTGGTCTCAAGTCAGTGTACAGGTAA), CC chemokine ligand (CCL)5 (formerly called RANTES; 5'-CGCTGTCATCCTCATTGCTA, 3'-GCTGTCTCGAACTCCTGACC), CCL2 (formerly called MCP-1; 5'-GCCTCCAGCATGAAAGTCTC, 3'-TAAAACAGGGTGTCTGGGGA), IL-1β (5'-TGCACGATGCACCTGTACGA, 3'-AGGCCCAAGGCCACAGGTAT), IL-6 (5'-GTTCCTGCAGAAAAAGGCAAAG, 3'-CTGAGGTGCCCATGCTACATTT), matrix metalloproteinase (MMP)-3 (5'-ATGGAGCTGCAAGGGGTGAG, 3'-CCCGTCACCTCCAATCCAAG), and vascular endothelial cell growth factor (VEGF) (5'-ATTGGAGCCTTGCCTTGCTG, 3'-CCAGGGTCTCGATTGGATGG). The cycling program was 94°C for 1 minute, 62°C for 1 minute, and 72°C for 1 minute for 25 or 30 cycles, followed by a final extension for 1 minute. The PCR products were electrophoresed on 1.5% agarose gel and stained with ethidium bromide. The relative intensities of the bands were quantified using image analysis software (NIH Image version 1.63; National Institute of Health, Bethesda, MD, USA).

### ELISA

RA-FLS were cultured and starved similarly as described above except 24-well culture plates were used. Twenty minutes after addition of DHMEQ (10 μg/ml) or vehicle, TNF-α (5 ng/ml) was added and 24 hours later the supernatant was harvested and kept at -20°C until use. ELISA kits for CCL2, CCL5, IL-1β and IL-6 were purchased from BioSource (Camacillo, CA, USA), and that for MMP-3 was from MBL (Nagoya, Japan).

### Flow cytometry

RA-FLS were cultured and starved as described above except 60 mm culture dishes were used. Twenty minutes after addition of DHMEQ (10 μg/ml) or vehicle, TNF-α (5 ng/ml) was added and 14 hours later the cells were washed with PBS, detached by trypsin-EDTA, and suspended in PBS. The prepared cells were incubated with monoclonal antibody to intercellular adhesion molecule (ICAM)-1 (BD Sciences, San Jose, CA, USA), vascular cell adhesion molecule (VCAM)-1 (BD Sciences), or isotype-matched mouse IgG for 20 minutes, followed by a detection with phycoerythrin-conjugated goat anti-mouse IgG (Southern Biotechnology Associates, Birmingham, AL, USA), and analyzed with an Epics XL flow cytometer (Beckman Coulter, Miami, FL, USA).

For detection of apoptotic cells, cells were prepared as above, DHMEQ (0, 5, or 10 μg/ml) or 1 μmol/l staurosporin (Wako, Osaka, Japan) was added, the cells were stimulated with TNF-α (5 ng/ml) 20 minutes later, further incubated for 14 hours, and stained with Cy3-labeled annexin V (MBL).

### Proliferation assay

RA FLS were cultured at a density of 10^4^/well in 96-well culture plates for 18 hours and then starved for 24 hours in Dulbecco's modified Eagle's medium with 2 μmol/l 2-mercaptoethanol, without FCS. After serum starvation, 0–10 μg/ml DHMEQ was added followed 20 minutes later by addition of 5 ng/ml TNF-α and 5 μCi/ml [^3^H]thymidine. The cells were further cultured for 48 hours and incorporation of ^3^H during the last 24 hours was measured in a scintillation counter.

### Electrophoretic mobility shift assay

RA FLS were cultured, starved, and stimulated as described above for RT-PCR, and nuclear extracts were prepared using NucBaster Protein Extraction Kit (Novagen, Darmstadt, Germany). The protein concentrations of the extracts were estimated by BCA Protein Assay Kit (Pierce, Rockford, IL, USA), and the extracts were kept at -80°C until use. ^32^P-labeled oligonucleotide containing the NF-κB binding sequence (5'-AGTTGAGGGGACTTTCCCAGGC-3') was used as a probe. Ten micrograms of the nuclear extract was incubated with 2 μg poly(dI-dC) for 30 minutes at room temperature in 20 μl reaction buffer containing 20 mmol/l HEPES, 20% glycerol, 100 mmol/l KCl, and 0.2 mmol/l EDTA, at pH 7.9. Following incubation, ^32^P-labeled probe was added to the mixture with or without 100-fold excess unlabeled oligonucleotide as a competitor and incubated for a further 30 minutes at room temperature. The protein-DNA complexes were separated from the free probe by 4% PAGE. For supershift assays, 1 μg of antibody to p50, p52 (Santa Cruz Biotech, Santa Cruz, CA, USA), or p65 (Chemicon, Temecula, CA, USA) was added to the sample and incubated for 30 minutes at 4°C before electrophoresis.

### Statistical analysis

Results were compared using two-sided, unpaired Student's t-tests.

## Results

### Therapeutic effect of DHMEQ on collagen-induced arthritis

The *in vivo *anti-inflammatory effect of DHMEQ was first demonstrated in a type of CIA model described by Matsumoto and coworkers [21], in which they showed a prophylactic effect of this compound when administered at 2–4 mg/kg, three times a week, from the day of booster immunization, although the CIA protocol was different from that in the present study and they did not use adjuvant. To test the therapeutic effect on a standard CIA model, we included only those mice that had apparently begun to develop arthritis by day 5 after the booster immunization with collagen and complete adjuvant, and started therapy with DHMEQ at 100 μg (5 mg/kg) daily.

**Figure 1:**
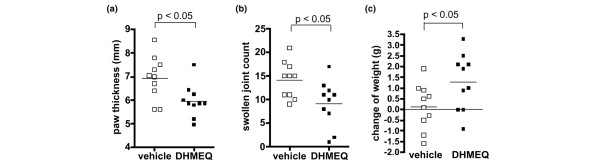
Clinical effect of NF-κB inhibitor DHMEQ on collagen-induced arthritis in DBA/1J mice. After onset of arthritis, animals were treated with 100 μg/day dehydroxymethylepoxyquinomicin (DHMEQ; ■; *n *= 10) or vehicle (□; *n *= 10). **(a) **Sum of the thickness of the right and the left hind paws of each mouse after 2 weeks of treatment. Each paw was measured twice and the average was plotted. **(b) **Sum of the number of swollen joints (described in the Materials and methods section) in the right and the left hind paws of each mouse after 2 weeks of treatment. Each paw was counted twice and the average was plotted. Maximum possible number is 32 per mouse. **(c) **Change in body weight of each mouse during the first week of treatment. Horizontal bars represent the mean. NF-kB, nuclear factor-κB.

**Figure 2:**
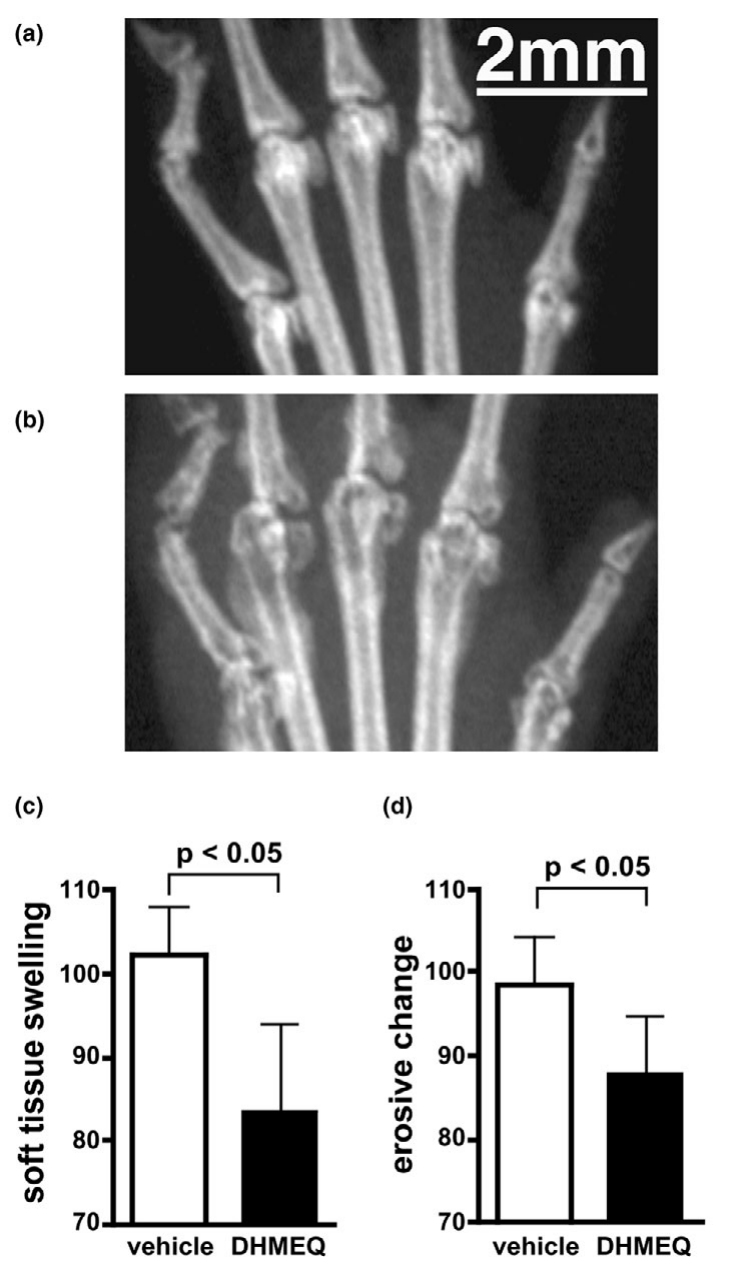
Effect of DHMEQ on radiographic findings in collagen-induced arthritis in mice. **(a) **A representative radiograph of the left metatarsophalangeal (MTP) joints of a mouse treated with dehydroxymethylepoxyquinomicin (DHMEQ), which shows small bone erosions, and **(b) **that of a control mouse, which shows remarkable soft tissue swelling and large bone erosions. **(c) **Soft tissue swelling and **(d) **bone erosions of bilateral second, third and fourth MTP joints observed in the radiographs were scored as described in the Materials and methods section. Values are expressed as the mean ± standard deviation of the total scores of 10 mice in each group, determined by five independent observers.

**Figure 3:**
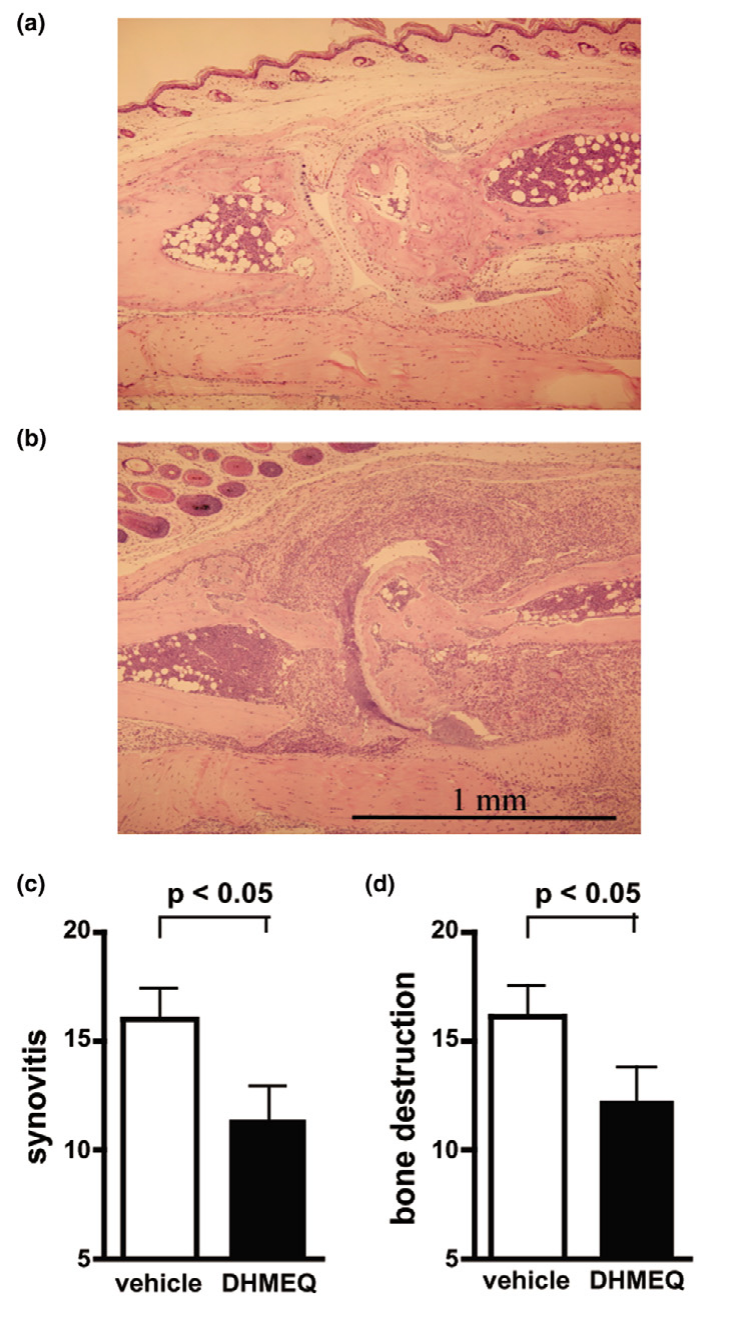
Effect of DHMEQ on histopathologic findings in collagen-induced arthritis in mice. **(a) **A representative specimen of the metatarsophalangeal (MTP) joint of a dehydroxymethylepoxyquinomicin (DHMEQ)-treated mouse, showing almost normal findings, and **(b) **that of a control mouse showing remarkable cell infiltration in the synovium and bone destruction accompanied by pannus invasion into the marrow space. The severity of **(c) **synovitis and **(d) **bone destruction in the specimens were scored as described in the Materials and methods section. Values are expressed as the mean ± standard deviation of the total scores of 10 mice in each group, determined by five independent observers.

After 14 days of therapy (from days 5 to 18), the thickness of the hind paws in the DHMEQ treated group (mean ± SD; 5.97 ± 0.66 mm) was significantly lower than that in the control group (6.95 ± 0.88 mm) that received vehicle alone (Fig. 1a). The number of swollen joints was also significantly lower in the DHMEQ-treated group (9.20 ± 4.64 versus 14.20 ± 3.68; Fig. 1b). During the first week of treatment, these young animals exhibited growth retardation, as estimated from their body weights, likely resulting from severe inflammation. However, this slow-down was significantly less in the DHMEQ group (weight gain from days 5 to 12; 1.29 ± 1.25 g) than in the control group (0.14 ± 1.01 g; Fig. 1c), suggesting that DHMEQ alleviated inflammation and was tolerable at the dose tested. Radiographs showed various degrees of soft tissue swelling and destructive changes in bone (Fig. 2a, b). The scores of these findings were both significantly lower in the group treated with DHMEQ (Fig. 2c, d). At the histologic level, various degrees of synovitis and bone destruction were observed (Fig. 3a, b). In severe cases, marked infiltration of mononuclear cells were present within the synovium, and pannus was frequently observed to penetrate into the bone marrow space. Again, DHMEQ reduced these findings significantly (Fig. 3c, d).

### Inhibition of nuclear factor-κB in rheumatoid arthritis fibroblast-like synoviocytes by DHMEQ

To examine the mechanisms underlying the antiarthritic effect of DHMEQ, as well as its effect on human cells, RA FLS lines were established from several patients with RA and used for the present experiments. We should like to note that it was previously confirmed that these cells expressed neither CD14 nor HLA class II [23], which means that they did not contain either macrophages or dendritic cells.

**Figure 4:**
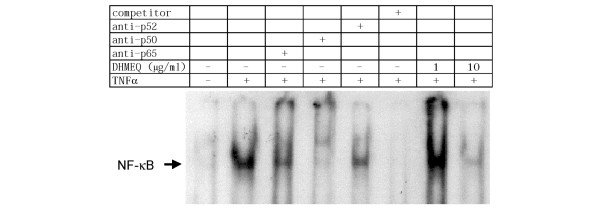
Inhibition by DHMEQ of NF-κB in rheumatoid arthritis fibroblast-like synoviocytes. Nuclear extracts were obtained from unstimulated and tumor necrosis factor (TNF)-α-stimulated rheumatoid arthritis (RA) fibroblast-like synoviocytes (FLS) and nuclear factor-κB (NF-κB) DNA-binding activity was examined by electrophoretic mobility shift assays. For supershift assays, the DNA-protein mixture was incubated with antibodies to p65, p50, or p52 before electrophoresis. To confirm the specificity of the assay, 100-fold excess of unlabeled NF-κB probe was included as a competitor. To assess whether dehydroxymethylepoxyquinomicin (DHMEQ) inhibits NF-κB activation in RA FLS, the cells were incubated with DHMEQ for 20 minutes before the stimulation with TNF-α. Data shown are representative of three independent experiments.

The effect of DHMEQ on NF-κB activation in RA FLS was examined using electrophoretic mobility shift assay (Fig. 4). Unstimulated RA FLS in serum-free medium exhibited only a faint band corresponding to NF-κB, but stimulation with 5 ng/ml TNF-α increased the intensity of the band dramatically. In supershift assays, anti-p50 antibody virtually abrogated the band of NF-κB, and anti-p65 antibody also remarkably diminished the intensity of the band. Anti-p52 antibody was much less effective, suggesting that the major components of the activated NF-κB in TNF-α-stimulated RA FLS were p65 and p50. An excess amount of unlabeled NF-κB probe abolished the band, confirming the specificity of this assay. When DHMEQ was added 20 minutes before the stimulation with TNF-α, the band representing NF-κB was abrogated almost completely at 10 μg/ml but not significantly at 1 μg/ml. Based on these findings, the following experiments were carried out using 10 μg/ml DHMEQ unless otherwise indicated.

### Suppression of inflammatory mediators by DHMEQ

Among the many molecules that are involved in inflammatory responses, a set of representative chemokines, ILs, MMP-3, and VEGF were selected to test the effect of DHMEQ. IL-6 is known to be an NF-κB-dependent cytokine [24,25] and is one of the key cytokines in RA pathogenesis, as evidenced by the fact that an anti-IL-6 receptor monoclonal antibody has been shown to reduce significantly RA disease activity in clinical trials [26]. We previously showed that chemokines CCL2 and CCL5 play a role not only in inflammatory cell migration but also in activation of RA FLS in an autocrine or paracrine manner [23]. Other investigators have shown that an antagonist to CCL2 suppressed arthritis in a murine model [27]. MMP-3 is among the cartilage-degrading enzymes and is known to be regulated by the NF-κB pathway in RA synovium [28]. VEGF is one of the angiogenic factors that are involved in the neovascularization in RA joints [29].

**Figure 5:**
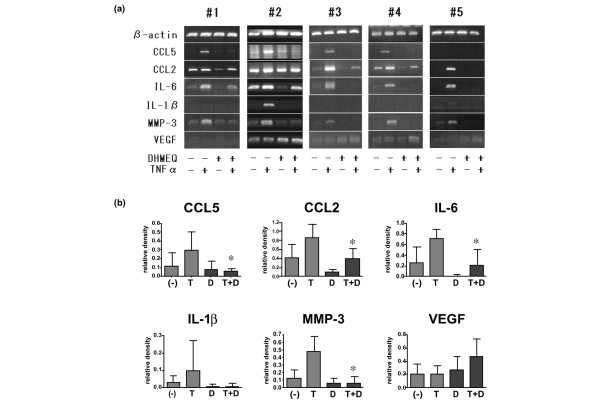
Effect of DHMEQ on inflammatory mediator mRNA expression by RA FLS stimulated with TNF-α. **(a) **Five rheumatoid arthritis (RA) fibroblast-like synoviocyte (FLS) cell lines (#1–#5) obtained from different patients were stimulated with tumor necrosis factor (TNF)-α in the presence or absence of 10 μg/ml dehydroxymethylepoxyquinomicin (DHMEQ) and mRNA expression of CC chemokine ligand (CCL)2, CCL5, IL-6, IL-1β, matrix metalloproteinase (MMP)-3, and vascular endothelial cell growth factor (VEGF) was examined by RT-PCR. **(b) **Densitometric analysis of these results. Intensity of each band was normalized relative to that of β-actin in the same lane, and the mean ± standard deviation of the five cell lines are shown. *P < 0.05 versus T. D, DHMEQ; T, TNF-α.

The effect of DHMEQ on mRNA expression of these key molecules was first examined by RT-PCR. As shown in Fig. 5, there was some heterogeneity in the mRNA expression levels depending on the cell line used. However, mRNA levels of CCL2, IL-6, and MMP-3 tended to be consistently increased by TNF-α stimulation and suppressed by DHMEQ. CCL5 mRNA was not detected in one of the cell lines (#5), but in other cell lines it was enhanced by TNF-α and suppressed by DHMEQ. IL-1β mRNA was barely detectable in some of the cell lines tested, at least under these assay conditions. However, after TNF-α stimulation IL-1β mRNA was clearly expressed in cell line #2 and faintly in lines #1 and #5; in all cases the expression was diminished by treatment with DHMEQ. In contrast, the level of VEGF mRNA was neither significantly increased by TNF-α nor suppressed by DHMEQ. We applied a constant amount of RNA to each tube, and observed virtually constant intensity of β-actin mRNA, irrespective of treatment with DHMEQ; this suggested that this compound did not affect the housekeeping activity of the cells.

**Figure 6:**
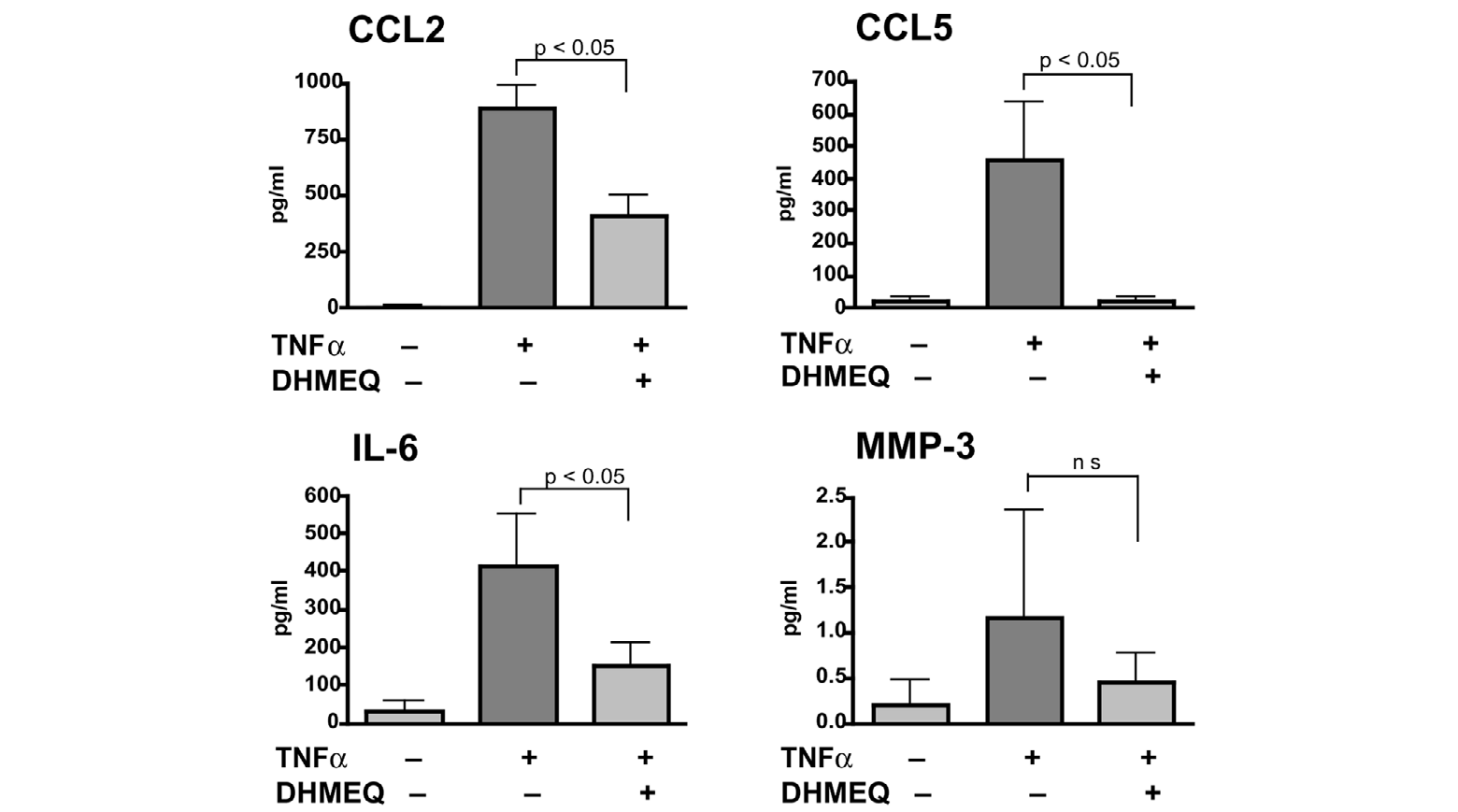
Suppressive effect of DHMEQ on inflammatory mediator production by RA-FLS at the protein level. Rheumatoid arthritis (RA) fibroblast-like synoviocytes (FLS) were stimulated with tumour necrosis factor (TNF)-α in the presence or absence of 10 μg/ml dehydroxymethylepoxyquinomicin (DHMEQ), and levels of secreted CC chemokine ligand (CCL)2, CCL5, IL-6, and matrix metalloproteinase (MMP)-3 in the culture supernatants were measured using ELISA. Values are expressed as the mean ± standard deviation of three independent experiments.

The suppressive effect of DHMEQ on the TNF-α-induced expression of CCL2, CCL5, IL-1β, IL-6 and MMP-3 was further tested at the protein level by ELISA (Fig. 6). CCL2, CCL5 and IL-6 were barely detected in the serum-free culture supernatant of the cells unless the cells were stimulated, but were produced abundantly after TNF-α stimulation. This production was significantly suppressed by DHMEQ. Similarly, production of MMP-3 tended to be suppressed by DHMEQ, although this was not statistically significant. Because the level of IL-1β was lower than the detection limit (10 pg/ml), even after stimulation with TNF-α, we could not confirm the effect of DHMEQ on IL-1β expression by ELISA.

### Suppression of adhesion molecule expression by DHMEQ

**Figure 7:**
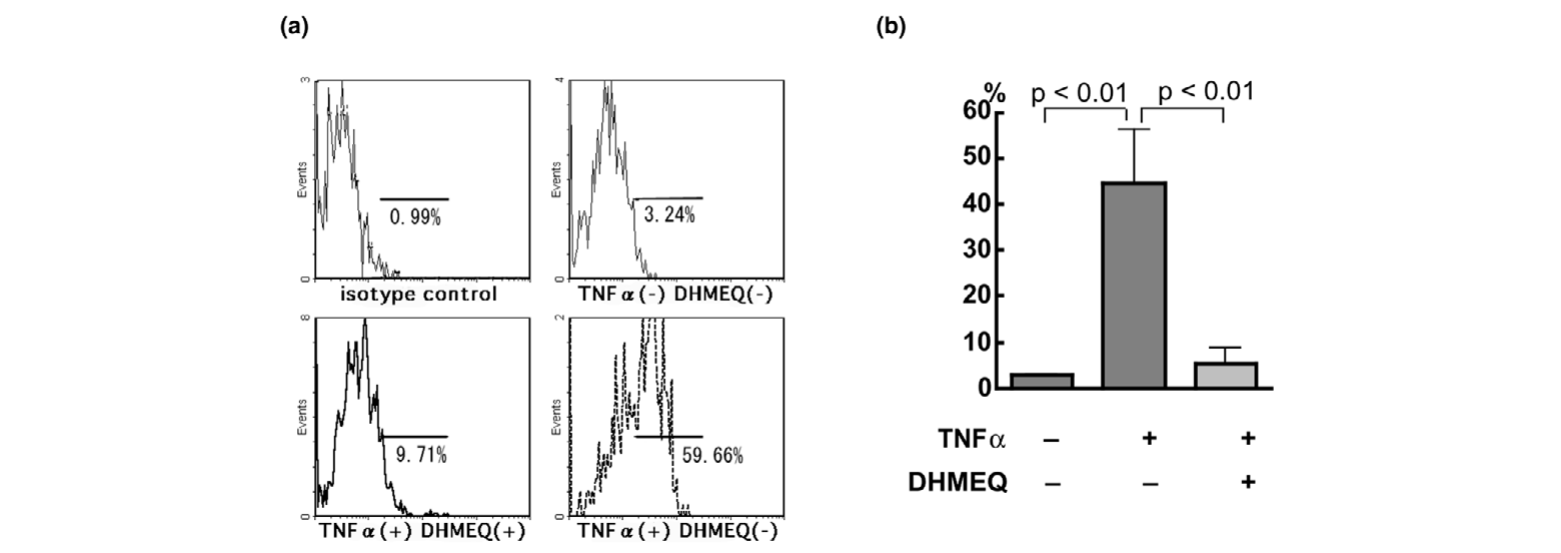
Suppression of ICAM-1 expression by DHMEQ. Shown, using flow cytometry, is suppression of intercellular adhesion molecule (ICAM)-1 expressed on tumor necrosis factor (TNF)-α-stimulated rheumatoid arthritis (RA) fibroblast-like synoviocytes (FLS) by dehydroxymethylepoxyquinomicin (DHMEQ). Cells were preincubated for 20 minutes with 10 μg/ml DHMEQ or vehicle. TNF-α stimulated or unstimulated RA FLS were incubated with isotype-matched control IgG or anti-ICAM-1 antibody, followed by phycoerythrin-labeled second antibody. **(a) **Representative data are shown, along with **(b) **the means ± standard deviation of ICAM-1-positive cells in four independent experiments.

**Figure 8:**
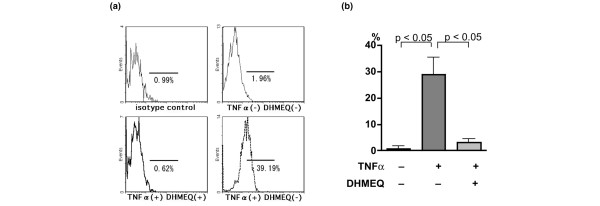
Suppression of VCAM-1 expression by DHMEQ. Suppression of vascular cell adhesion molecule (VCAM)-1 expressed on tumor necrosis factor (TNF)-α-stimulated rheumatoid arthritis (RA) fibroblast-like synoviocytes (FLS) by dehydroxymethylepoxyquinomicin (DHMEQ). Flow cytometric analysis was carried out (as in Fig. 7) except that anti-VCAM-1 antibody was used. **(a) **Representative data are shown, along with **(b) **the means ± standard deviation of VCAM-1-positive cells in three independent experiments.

Adhesion molecules ICAM-1 (CD54) and VCAM-1 (CD106) are expressed at higher levels in the synovial tissue of RA than in osteoarthritis [30,31] and are implicated in the interaction between leukocytes and RA FLS that contributes to the synovitis. Flow cytometric analysis showed that 2.7 ± 0.5% (mean ± standard deviation [SD] of four independent experiments) of RA FLS expressed ICAM-1 in serum-free medium, and the ratio of cells expressing ICAM-1 markedly increased up to 44.5 ± 11.7% after stimulation with TNF-α (Fig. 7). In the presence of DHMEQ, however, this ratio significantly decreased to 5.3 ± 3.4%. On the other hand, VCAM-1 was expressed on only 0.9 ± 1.0% (mean ± SD of three independent experiments) of the unstimulated cells, but 29.0 ± 11.2% of the cells expressed VCAM-1 after TNF-α stimulation (Fig. 8); this ratio significantly decreased to 3.1 ± 2.5% by the effect of DHMEQ.

### Suppression of proliferative activity of rheumatoid arthritis fibroblast-like synoviocytes by DHMEQ

**Figure 9:**
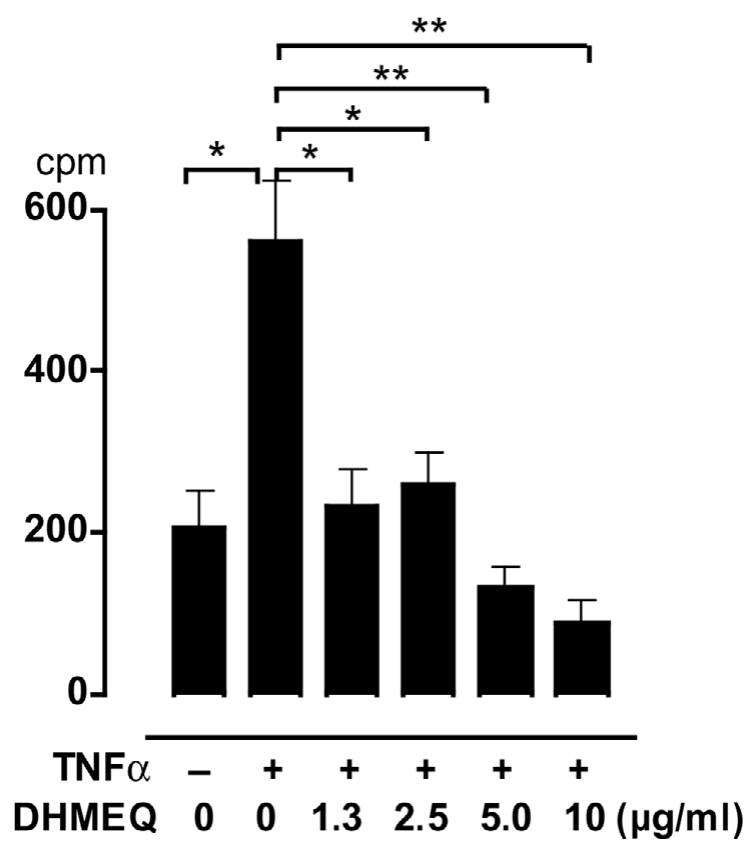
Suppression of proliferative activity of RA FLS by DHMEQ. Rheumatoid arthritis (RA) fibroblast-like synoviocytes (FLS) were stimulated with tumor necrosis factor (TNF)-α or unstimulated in the presence or absence of dehydroxymethylepoxyquinomicin (DHMEQ), cultured for 48 hours, and incorporation of [^3^H]thymidine during the last 24 hours was measured. Values are expressed as mean ± standard deviation of triplicate measurements. Data shown are representative of three independent experiments. *P < 0.01, **P < 0.001. cpm, counts/minute.

One of the prominent characteristics of RA FLS is their proliferative activity, which leads to pannus formation as well as swelling of the joints, and pathways controlling the proliferation of RA FLS include an NF-κB-dependent pathway [32]. Representative data shown in Fig. 9 indicate that RA FLS incorporate a certain amount of [^3^H]thymidine even without stimulation (mean ± SD; 207 ± 36 counts/minute), which was significantly higher than the background level (29 ± 1.2 counts/minute; *P *< 0.01). This moderate activity increased to 582 ± 53 counts/minute with stimulation with TNF-α, and DHMEQ significantly suppressed this proliferative activity in a dose-dependent manner. At 5.0 μg/ml or higher concentration of DHMEQ, proliferative activity of the cells was lower than that of unstimulated cells, suggesting that DHMEQ suppressed spontaneous proliferation as well as TNF-α-induced proliferation.

### Cytotoxic effect of DHMEQ on rheumatoid arthritis fibroblast-like synoviocytes

To test whether DHMEQ exhibits cytotoxicity at the concentration that suppressed inflammatory mediators, serum-starved RA FLS were further incubated for 14 hours after stimulation with TNF-α in serum-free medium with DHMEQ or the apoptosis inducer staurosporin. Thereafter, expression of annexin V binding phospholipid on the cell surface – an indicator of early phase of apoptosis – was measured using Cy3-labeled annexin V. With staurosporin, 10.1% of the cells were annexin V positive and the mean fluorescence intensity was 1.1 (Fig, 10). In contrast, in the presence of 5 and 10 μg/ml DHMEQ, the ratio and mean fluorescence intensity of annexin V positive cells remained less than 1% and 0.5%, respectively. Trypan blue dye exclusion test also showed virtually 100% viability of the cells incubated with DHMEQ (not shown).

## Discussion

Many stimuli are known to activate NF-κB, including TNF-α, IL-1β, anti-CD3 antibody (in T cells), oxidative stresses, viral products and lipopolysaccharides, and these act by means of protein kinases that phosphorylate IκB, leading to degradation of IκB by the proteasome and passage of NF-κB into the nucleus [2]. NF-κB regulates the expression of many genes that are involved in inflammatory responses, including TNF-α, IL-1β, IL-6, CCL2, CCL5, MMP-3, ICAM-1, VCAM-1, inducible nitric oxide synthase, and cyclo-oxygenase-2, all of which are known to participate in the pathogenesis of RA. Products of these genes coordinately enhance inflammatory reactions resulting in further activation of NF-κB. In fact, NF-κB components p50 and p65 were demonstrated to be activated in both macrophage-like and fibroblast-like synoviocytes as well as vascular endothelial cells in RA-derived synovial tissue but not in normal synovium [4-7]. In RA synovium p50 and p65 expression increases, especially at sites adjacent to the cartilage-pannus junction, and is thought to be implicated in cartilage destruction [33]. It is clear, therefore, that NF-κB is an important target molecule for RA therapy. Aspirin, sodium salicylate, corticosteroids, sulfasalazine, and gold salts were demonstrated, at least in part, to exhibit their activity by way of NF-κB suppression [34-37], and novel agents that are more specific to the NF-κB pathway than these classical agents – and are less costly than the recently marketed biologics – would be of great value. Indeed, inhibition of the NF-κB pathway by gene therapy [8-11] or by small molecular weight compounds [13-18] has recently been tested in experimental models of arthritis, demonstrating the efficacy of this strategy.

We previously showed that DHMEQ does not inhibit phosphorylation and degradation of IκB, but inhibits nuclear transport of p65 in TNF-α-stimulated COS-1 cells transfected with the DNA that encodes p65 combined with green fluorescent protein [20]. It does not affect TNF-α-induced activation of JNK, or nuclear transport of Smad2 or the large T antigen. This molecule should therefore be considered a unique inhibitor of NF-κB that acts at the level of nuclear translocation. This specificity is an advantage of DHMEQ over the inhibitors of the upstream molecules of the NF-κB pathway, because kinase inhibitors or proteasome inhibitors may suffer from disadvantages relating to specificity and undesired effects that are unrelated to the NF-κB pathway. Another advantage of DHMEQ over gene transfer methods is its simplicity of administration. In our model of mouse arthritis, subcutaneous daily injections of 5 mg/kg DHMEQ resulted in a significant therapeutic effect on arthritis. Although the efficacy of other administration protocols and the pharmacokinetics of DHMEQ remain to be studied in detail, small, cell-permeable compounds appear to have fewer obstacles to be overcome in comparison with gene transfer strategies because RA is a chronic systemic inflammatory disorder.

As a first step toward application in human cells, we tested the effect of DHMEQ on the function of RA synovial cells in culture. Thus far, studies that showed effects of NF-κB inhibitors using human synovial cells have been limited. However, suppression of expression of the key inflammatory cytokine IL-6, chemokines CCL2 and CCL5, matrix-degrading enzyme MMP-3, and adhesion molecules ICAM-1 and VCAM-1, as well as proliferative activity of the cells, suggested that DHMEQ may be efficacious in the treatment of RA synovitis. In the electrophoretic mobility shift assay, 10 μg/ml DHMEQ nearly completely inhibited NF-κB activity of the TNF-α-stimulated RA FLS, but its effect was not significant at 1 μg/ml (Fig. 4). Nevertheless, in the proliferation assay 1.3 μg/ml DHMEQ significantly suppressed TNF-α-stimulated thymidine uptake by RA FLS (Fig. 9). Variation is to a certain extent inevitable in experiments using RA FLS, but this discrepancy was reproducible. It seems possible, in assays that require longer incubation of cells, that secondary effects of NF-κB inhibition resulting from suppressed expression of cytokines and other regulatory molecules of cellular activity may merge with the direct effect of DHMEQ.

VEGF is thought to promote angiogenesis and enhance vascular permeability in inflamed tissue, but we did not observe suppression of VEGF mRNA expression by DHMEQ. A recent investigation showed suppression of IL-6-induced VEGF production by fibroblasts using a JNK inhibitor, which suggests that expression of VEGF is predominantly regulated not by the NF-κB pathway but by the activator protein-1 pathway [38]. In this regard, it was reported that cyclosporin A, which is a widely used immunosuppressant and is effective to some degree in RA, suppressed expression of VEGF by RA-FLS by way of suppressing activator protein-1 binding activity [29].

The most important goal in RA therapy is the prevention of bone destruction in order to maintain normal function of the joints. It was recently demonstrated that DHMEQ suppresses osteoclastogenesis in a culture system of mouse bone marrow derived macrophage precursor cells stimulated with RANKL (receptor activator of NF-κB ligand) and macrophage colony-stimulating factor, and suppresses the bone-resorbing activity of mature osteoclasts [39]. Therefore, it is of interest to study further the *in vivo *effect of this inhibitor on bone-resorbing activity in severe arthritis.

Along with efficacy, safety issues should be addressed. In the present study we observed no abnormality in the behavior of the mice treated with DHMEQ; rather, they exhibited less weight loss than did the control mice during the active period of inflammation. In *in vitro *experiments, annexin V staining and trypan blue staining confirmed that cell viability did not decrease. The mRNA expression of VEGF and β-actin was not affected by DHMEQ. However, NF-κB is known to play a role in preventing cell apoptosis [40]. Massive hepatocyte apoptosis in p65-deficient mice is an extreme example of the antiapoptotic role of NF-κB [41]. We also observed significant apoptosis in some tumor cells transplanted into nude mice treated with DHMEQ (8 mg/kg per day, by intraperitoneal injection), but the mice did not exhibit adverse effects [42]. The relationship between the anti-inflammatory effect of DHMEQ and apoptosis of cells in inflamed as well as normal tissue remains to be further examined.

## Conclusion

We showed herein that an NF-κB nuclear translocation inhibitor DHMEQ had a therapeutic effect on CIA in mice, and suppressed the expression of inflammatory molecules and the proliferative activity of TNF-α-stimulated RA FLS. Although its effect on other human cell types, especially T cells, vascular endothelial cells and osteoclasts, are currently under investigation, these findings suggest that FLS are among the important targets on which DHMEQ exerts its antiarthritic effect. This agent may be a promising candidate for further clinical development.

## Abbreviations

CCL = CC chemokine ligand; CIA = collagen-induced arthritis; DHMEQ = dehydroxymethylepoxyquinomicin; DMSO = dimethyl sulfoxide; ELISA = enzyme-linked immunosorbent assay; FCS = fetal calf serum; FLS = fibroblast-like synoviocyte; ICAM = intercellular adhesion molecule; IκB = inhibitor of NF-κB; IL = interleukin; JNK = c-Jun N-terminal kinase; MMP = matrix metalloproteinase; MTP = metatarsophalangeal; NF-κB = nuclear factor-κB; PBS = phosphate-buffered saline; RA = rheumatoid arthritis; RT-PCR = reverse transcriptase-polymerase chain reaction; SD = standard deviation; TNF = tumor necrosis factor; VCAM = vascular cell adhesion molecule; VEGF = vascular endothelial cell growth factor.

## Competing interests

The authors declare that they have no competing interests.

## Authors' contributions

KW carried out *in vitro *experiments. TN and TK designed the study, carried out *in vivo *experiments, and drafted the manuscript. NM collected the clinical materials and revised the manuscript. KU synthesized a critical chemical. All authors read and approved the final manuscript.

## Figures and Tables

**Figure 10:**
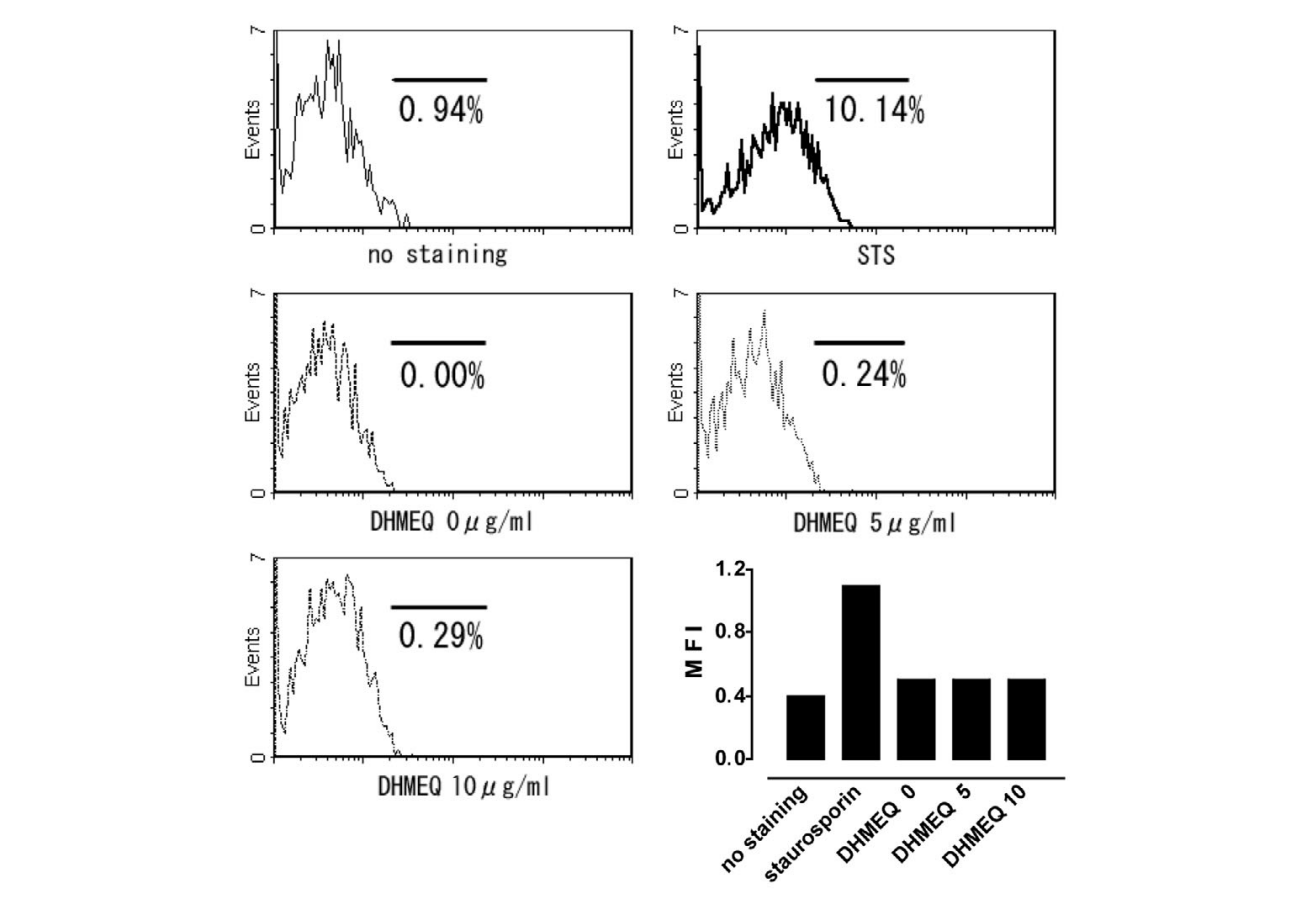
Cytotoxicity of DHMEQ. Significant cytotoxicity was not observed in rheumatoid arthritis (RA) fibroblast-like synoviocytes (FLS) treated with dehydroxymethylepoxyquinomicin (DHMEQ). Cells were stimulated with 5 ng/ml tumor necrosis factor (TNF)-α and incubated in serum-free medium for 14 hours with 0–10 μg/ml DHMEQ or with the apoptosis inducer staurosporin (1 μmol/l), and Cy3-labeled annexin V binding cells were measured by flow cytometry. Data shown are representative of three independent experiments. MFI, mean fluorescence intensity.
